# Living with cryptoglandular anal fistula: a qualitative investigation of the patient's experience through semi-structured patient interviews

**DOI:** 10.1007/s11136-022-03098-y

**Published:** 2022-02-17

**Authors:** Nusrat Iqbal, Astrid J. H. M. Machielsen, Stephanie O. Breukink, Rebecca Woodcock, Gillian Kane, Laith Alrubaiy, Ugo Grossi, Merel L. Kimman, Phil J. Tozer

**Affiliations:** 1 Robin Phillips’ Fistula Research Unit, St Mark’s Hospital, London, UK; 2 Department of Surgery and Cancer, Imperial College London, London, UK; 3 Maastricht University Medical Centre, Maastricht, The Netherlands; 4 Department of Surgery and Colorectal Surgery, Maastricht University Medical Centre, Maastricht, The Netherlands; 5 Nutrim School of Nutrition and Translational Research in Metabolism, Maastricht University Medical Centre, Maastricht, The Netherlands; 6 Grow School for Oncology and Developmental Biology, Maastricht University Medical Centre, Maastricht, The Netherlands; 7 London, UK; 8 Belfast, UK; 9 Department of Gastroenterology, St Mark’s Hospital, London, UK; 10 Tertiary Referral Pelvic Floor and Incontinence Centre, Regional Hospital, Treviso, Italy; 11 Department of Clinical Epidemiology and Medical Technology Assessment, Care and Public Health Research Institute (CAPHRI), Maastricht University Medical Centre, Maastricht, The Netherlands

**Keywords:** Anal fistula, Quality of life, Qualitative research

## Abstract

**Purpose:**

Cryptoglandular anal fistula continues to be a subject of extensive surgical research due to the lack of effective and enduring treatments, some of which incur risks to continence and quality of life. However, the patient experience of disease has seldom been reported. The aims of this study are to understand the impact of living with a fistula and the treatment outcomes that are valued by patients.

**Methods:**

Patients with cryptoglandular anal fistula were recruited using purposive sampling from two tertiary referral centres in the UK and the Netherlands. Patients underwent semi-structured interviews that were audio-recorded and transcribed verbatim. Dutch transcripts were translated into English and underwent independent, thematic analysis using open coding by two study team members to identify common themes and sub-themes.

**Results:**

Twenty interviews were conducted before saturation was reached (11 male, median age 49, Interquartile range 39–55 years). Four broad themes emerged, covering the physical symptoms of fistula, the patient journey towards understanding the condition, life impact, and treatment. Several inter-related sub-themes were found, reflecting the extensive impact and adjustment that the disease entails.

**Conclusion:**

The impact of cryptoglandular anal fistula extends beyond the physical symptoms of pain and discharge, requires significant readjustment, and often negatively impacts psycho-social wellbeing. These aspects of disease should receive greater attention in future assessment of treatment and quality of life.

**Supplementary Information:**

The online version contains supplementary material available at 10.1007/s11136-022-03098-y.

## Plain English summary

A cryptoglandular anal fistula is a small tunnel connecting the inner surface of the anus or rectum to the overlying skin, which happens as a result of chronic infection and can be difficult to treat. Patients typically suffer from pain and continuous discharge which can greatly impact quality of life. Despite this, few studies have conducted a detailed investigation into what it is like to live with a fistula. We conducted interviews with fistula patients in the UK and Netherlands and found that the effects were broad, influencing work, relationships, daily life and activities and there were certain aspects of medical care that patients found challenging. This has implications for future assessment of fistula, which should expand beyond the focus on physiological and clinical symptoms and should incorporate those outcomes that are important to patients.

## Introduction

An anal fistula is an abnormal epithelialised connection between the anal canal and the perianal skin, affecting 1–2 people per 10,000 per year [1]. Most cases occur due to an infection of the mucus glands lining the anal canal, which discharges via the perianal skin. Chronic, ongoing infection of this pathway leads to the formation of the epithelialised fistula tract, as described by the cryptoglandular hypothesis [2].

Most of the research into cryptoglandular anal fistula is concerned with evaluating the physiological and clinical outcomes of surgical treatment [3] including healing and continence. However, anal fistulae have a frequent and profound negative impact on quality of life (QoL) [4]. Patients typically experience perianal pain and ongoing discharge from the external opening, in addition to infective episodes and the development of recurrent abscesses requiring surgical drainage. These symptoms drive a significant QoL burden and require considerable practical adjustments [4–7]. When asked about the most important QoL issues associated with fistula treatment, patients were more likely to prioritise factors relating to psycho-social wellbeing [8]. In contrast, surgeons and most of the known literature focussed primarily on the impact of physical symptoms [8]. Surgical treatment as been demonstrated to improve QoL [6], even in patients who see a minor deterioration in continence as a result [9, 10].

Despite patient prioritisation of the psycho-social impact of a fistula [8], only a minority of interventional studies measure this as an outcome of treatment [3]. To our knowledge, a detailed assessment of the reality of living with an anal fistula is seldom reported in the literature. Recently, qualitative exploration of patient experience in those with an anal fistula secondary to Crohn’s disease has found that the burden of disease has far-reaching consequences for emotional, physical and social wellbeing [11]. It is unknown whether similar themes can be found in those with a cryptoglandular anal fistula, however, given that the majority of fistulae have a cryptoglandular aetiology, this is an essential area of research.

The aim of this study is to further understand the impact of cryptoglandular anal fistula on patient quality of life through a detailed, qualitative investigation of the patient experience.

## Methods

This qualitative study is reported according to the Standards for Reporting Qualitative Research (SRQR) [12]. Appropriate ethical approval was obtained in the UK (REC reference 19/WM/0296) and the Netherlands (METC 2018-0913).

### Qualitative approach and research paradigm

We aimed to conduct an exploration of patient experience of anorectal fistula by conducting semi-structured qualitative interviews. A pragmatic constructivist approach was used to investigate the subjective experiences of anal fistula patients, without assuming a single objective reality [13].

### Researcher characteristics

Interviews were conducted by clinician–researchers with experience in the assessment of patients with anal fistula. In order to minimise biases in questioning and interpretation, interviewers were not known to, nor had any pre-existing clinical relationship to the participants. Interviewers were trained and supervised by qualitative researchers on the study management team who also oversaw data analysis and interpretation. Furthermore, in order to minimise the influence of interviewer’s clinical backgrounds on the line of questioning and collection of data, interview guides and coding strategies were reviewed by qualitative researchers and patient representatives prior to implementation and analysis.

### Context

Patients were recruited from outpatient clinics at tertiary referral centres in the UK and the Netherlands. This provided an opportunity to recruit and interview patients during a period of relative symptom and disease stability, in that they had not been subjected to any recent emergency intervention or acute deterioration of symptoms that may have influenced study results.

### Sampling strategy and ethical issues

Participants were recruited by clinician-researchers who later conducted the interviews. Patients aged 18 years and older with a new or previously treated anal fistula not known to have any underlying aetiology were approached in the outpatient clinic and offered written study information, a consent form, and contact details of the researcher so that they could address any queries arising from the participant information sheet. Potential participants were encouraged to take the information away to read fully prior to returning the completed consent form if they wished to participate, however some provided informed consent within the same clinical care episode. Purposive sampling was used to ensure the selection of patients who had just been referred and were therefore earlier into their treatment journey, as well as those with longstanding symptoms, aiming for equal numbers of male and female participants ranging in geographical location. The sample size was determined by the point at which saturation was reached, identified when no new themes emerged from three consecutive interviews.

### Data collection methods, instruments and data processing

As patients attending tertiary centres were travelling from geographically distant sites, interviews were conducted via telephone from 1 to 31st December 2019. An interview guide was developed by the study management team and approved by patient representatives (Online Appendix 1). This was designed to cover all aspects of the disease process, from early symptoms and diagnosis, to post-treatment adjustments and desired outcomes. The interview guide was largely used to provide prompts for discussion, with deeper questioning and exploration of the issues raised by participants made by the interviewers where possible. All interviews were audio-recorded on to devices used specifically for study purposes. Prior to starting the recording, interviewers confirmed consent for participation and advised participants to avoid quoting identifiable information during the course of the interview, in order to maintain participant anonymity. All interviews were transcribed verbatim. Dutch transcripts were translated into English using the translation function in Microsoft Word. The accuracy was assessed by bilingual members of the study management team, who reviewed both Dutch and English transcripts as well as the subsequent coding of interviews by referring to the original transcripts. Interview translation was found to be accurate with no apparent loss of data identified between the original and translated manuscripts.

### Data analysis

Given the semi-structured nature of the interviews and the aim of obtaining a descriptive overview of patient experience, the framework method was used to conduct thematic analysis of transcribed interviews [14]. Open coding with an inductive approach was used, where data were collected and analysed outside of any pre-existing theoretical framework given the lack of detailed qualitative exploration in this field. Two researchers independently coded the first three interviews, after which members of the study team convened. Coding was compared, and preliminary categories were identified. Subsequent transcripts were then indexed using these codes and categories, which were charted into a matrix using Microsoft Excel. Within and between case analyses were conducted and overall themes were developed from the initial coded categories. Credibility of data analysis was enhanced by having two members of the study management team coding three interviews and comparing coding between reviewers. Member checks of the analysed interviews were not performed due to the time investment this would have required from the participants.

## Results

**Fig. 1:**
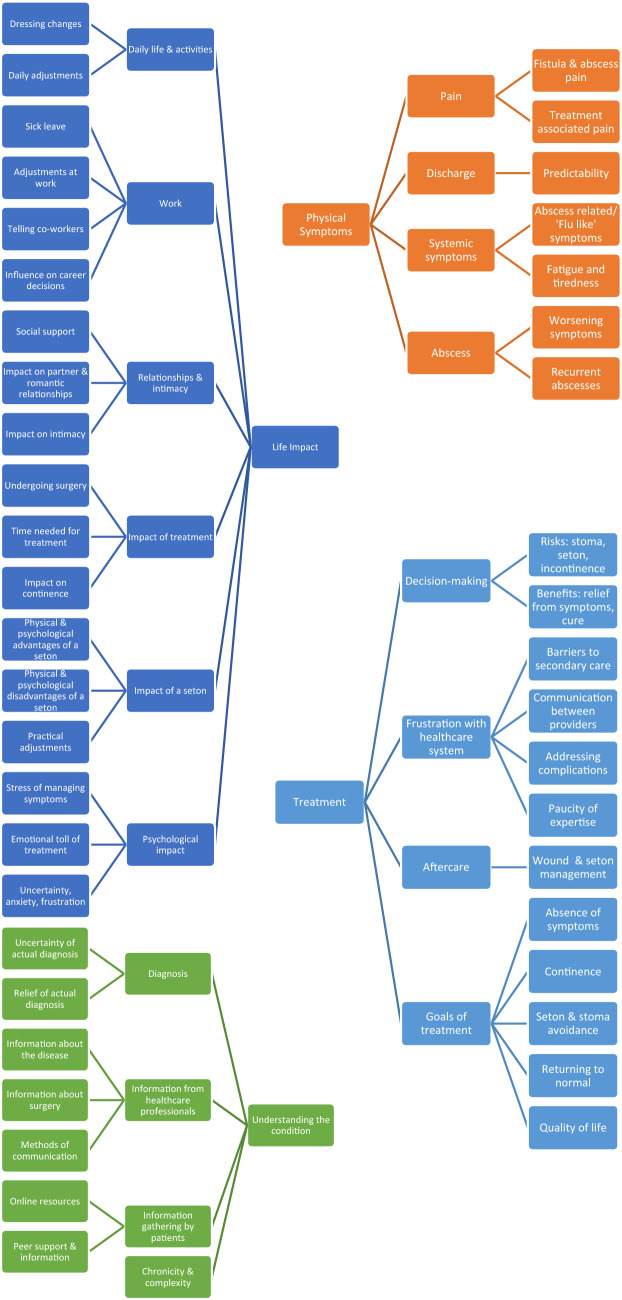
Central themes and sub-themes deduced from patient interviews regarding experiences of living with cryptoglandular anal fistula

Interviews were conducted with twenty patients (11 male, median age 48.5 Interquartile range 38.5–54.5) (Table 1), all patients were at various stages of treatment. Interview duration ranged from 23 to 72 min. Categories were organised into four broad themes, shown in Fig. 1, with illustrative quotes detailed in Table 2.

**Table 1:** Participant demographics

Demographics	Number of participants	
	UK	NL
Gender		
Female	5	4
Male	5	6
Age range		
18–30 years	0	1
31–40 years	5	1
41–50 years	3	2
51- 60 years	1	4
≥ 61 years	1	2
Duration of diagnosis		
0–24 months	6	4
25–48 months	1	2
> 48 months	3	4
Number of previous procedures		
0–1	6	6
2–4	4	4
> 4	0	0

**Table 2:** Interview themes and sub-themes with illustrative quotes

Theme	Subtheme	Quote
Physical symptoms	Pain	“It was very painful on and off. And it was debilitating in that it stopped you from, like I say, sitting for periods of time or walking far or whatever” UK 4
		“Pain I can't say like that, more uncomfortable. Pain has to be more intense. It was uncomfortable in itself. It was always there…” NL 1
	Discharge	“Sometimes I get loads and loads of drainage and then another day I might not have a lot but then I’ll be agony that night and then the next morning I’ve got loads again. So I don’t know if it’s where it’s starting to build up and then comes back down or what but yeah sometimes I remember in the early days thinking I would rather have an abscess than this because I remember feeling well this is just awful.” UK 10
	Systemic symptoms	“So, initial stages were just I’d got a fever type thing so high temperature, just feeling generally tired and unwell.” UK 7
		“Restless, no energy, sometimes I had a fever.” NL 3
	Abscess	“So that part, the whole thing was constantly abscessing so that was a problem that give me so much pain like I said in the cycle of three months. For two, three weeks I just can’t do anything. I have to have another surgery to have the abscess drained.” UK 2
		“Well, that's going to heal, and then another abscess came and that's been repeated a number of times.” NL 1
Understanding the condition	Diagnosis	“Not at first, he thought it might be a hair follicle that's a little irritated and we actually kept it on that as well. He said, "I can't find anything serious." But at some point, you feel, something's not right. It must be something else, so you'll tell me to go to the hospital for, yes, how do you say that, a second opinion.” NL 2
		“It felt like I was really… In a way I was relieved that I finally knew what was happening and for once I felt like he was saying to me, it’s this, it’s connected to there, we need to do this to drain it. And I remember sat there thinking oh somebody actually knows what it is now.” UK 10
	Information from healthcare professionals	“But the surgeon who was dealing with me she was very much of the philosophy of don’t worry about it, I don’t want you to think about it too much we need to deal with this a stage at a time. So, it wasn’t really… And I wasn’t really truly aware of exactly what was happening.” UK 1
		“And then it was just being provided with the information that was specific to me made a huge difference. So it wasn’t just like, oh, here’s a leaflet on whatever. It was like, here is your MRI. Let me show you what I mean by this. Let’s, you know. So it was the information about your own body and being given the options and having the time to actually discuss that and ask questions.” UK 4
		“And I came home the next day, still not really knowing what they did, what they didn’t do, what the outcome is, etc. So, I was left a little bit in the dark…. They talk to you when you're still drugged up. When you come back around a day later and that, you can’t remember a thing, you can’t remember anything they said.” UK 9
		“Yes, I'm having surgery and two months later a check-up appointment wasn't scheduled until two months later. How it was. Not until two months later.” NL 5
	Information gathering by patient	“I mean I did Google a lot. I read a lot of papers so I know all my options. So actually I asked them can you offer the laser or…. and they said we just can’t do it. We don’t have those here locally. That’s it. That’s their answer.” UK 2
		“I think because if I hadn’t have been on the forums that I was on, I wouldn’t have known half of it and I do think if I’d have woken up suddenly with this seton and I would’ve felt very alone and I think I’d have been very scared. UK 10
	Chronicity and complexity of condition	“But I remember saying to her at one point quite quickly how long before she sorts this out. And she looked at me and she said it could be years. And I was like, oh, okay, I never realised there was something a little bit more complicated involved.” UK 1
Life impact	Impact on work	“I had to go to the doctor’s surgery all the time really so it stopped me going to work. I mean they were quite good about and I probably could have carried on. But I guess it was a trigger for me to leave, time’s up.” UK 3
		“It’s not the fistula impacts the job that I’m doing. It’s like the indirect consequences because I wasn’t 100% myself and the work was a bit demanding so there was misunderstanding in the workplace because it’s something like invisible. Invisible disabilities most people don’t understand it. Most people have never even heard of this word, right? So people don’t really understand what it did.” UK 2
		“…because I just happened to be at a job interview today, and I'm like, yes, what am I going to do now, I have to say I might get another operation? So I thought, no, I'll see if I get hired first. Those are things I find difficult” NL 10
	Impact on daily living and activities	“I had to be always very careful because of this discharge. I have to have some pads all the time and to be careful about how at work, not to be visible. And a lot of stuff, also to have all the time chance to go to the toilet. It was very tiring so I can take care of myself also in this way. It’s pretty difficult, I could say.” UK 5
		“It's what I say, you often don't dare to go away too long, because if I have stools then I also have to see that I clean that wound immediately, that there is no dirt in it, so it really affects my life I think.” NL 7
		“And day-to-day activities were quite challenging, like sitting for any kind of period of time was painful, driving, walking to an extent. But it seemed to kind of change every day. So it was quite a different thing. Some days it would be fine and other days it would be insanely painful. So it was quite a strange kind of scenario.” UK 4
		“You can't sit properly. You can't walk far. Your side doesn't work, you can't. So that has quite an impact. And when it pops open, the junk that comes out of there, yes, he'd be walking around with sanitary pads for a few months.” NL 5
	Impact on relationships and intimacy	“I’m lucky I have a very understanding group of friends, very understanding family. So they have actually been incredibly supportive and completely understand if I’ve cancelled something or if I’ve said whatever, which again I can imagine might not be the case for everyone…. What I do think now is that I find the longer this goes on the less people care. I know that sounds really awful. But I think, you know when something’s gone on for so long, it just kind of becomes… I suppose it becomes normal for them as well.” UK 4
		“And for me, our intimate relationship between us, I don’t like the fact that there's a bloody hole in the backside and a bloody thread hanging out it, do you know what I mean? It’s just quite off putting. But for me, it’s a bit of a frigging turnoff myself. And you got a flaming 4 × 4 bit of gauze stuck to your bottom. It’s a bit of a passion killer.” UK 9
	Impact of treatment	“The worst part, the most traumatic part for me has been especially those first few years has been the going to and from for daily dressings. That was horrendous because it was just… It was painful; you had to psyche yourself up for it. It was time to get to the appointments was really difficult because nobody would come out because I’m young and able. But trying to get the bus to the… It's not easy.” UK 7
		“…so I can stand when I want to, but I don't always, because I've had surgery a number of times, [I can’t] control the exit, I'm not sure how else to say that. So the moment I think: I have to break wind, then I'm very afraid that I can't control that.” NL 10
	Impact of seton	“I had quite a difficult illness and I was a little bit paranoid that is there a build-up, is there a build-up. Once the seton was in I suppose it did allay those fears because obviously daily you see pus coming out so you know it’s not building up as such. You know it’s making its way out. So, I suppose, yes, once the seton was in I probably was a little bit more psychologically comfortable or whatever, less paranoid that I was going to become ill.” UK 1
		“Because the seton sounds normal to a colorectal surgeon but to normal people who haven’t really been sick before that’s really a big thing. It’s like oh this is so terrible but I really wanted to get rid of that seton.” UK 2
		“There was always something of caution, with the seton that it couldn't get out, or that I kept hooking in there with paper. I did, but that's an inconvenience for me.” NL 1
	Psychological impact	“So I think there’s that mental side as well where it’s challenging when you’re in pain regularly. You don't know what it is. Nobody seems to be doing very much. No one seems to be able to help you. Nothing you take helps. So it feels a little bit hopeless. Yes, which is, yes, quite hard.” UK 4
		“I managed to have a normal life, to be honest. It’s just more psychologically damaging than physically…. It’s something you live with constantly. You need to change the… You know, like I have a little gauze or whatever; you need to change it. It’s discharging disgusting fluid that smells disgusting. It affects your sexual life. It also affects your everyday… Like that consciousness of having something open, like a wound open, near your vagina and near your anus is quite intimate, but you feel it’s there; it’s a constant reminder that you’re not well there.” UK 8
		“Because, actually, the shock of then hearing I’ve just been through this quite traumatic process and now you’re telling me it hasn’t worked and I’m still where I was before except now, I still have an open wound was not great. Mentally, emotionally and, clearly, physically, I wasn’t in a great situation.” UK 4
		“…whether it's completely gone. I don't have that certainty. And since they didn't find an opening last time, I'm afraid it's going to happen again. And I just haven't been helped after opening it.” NL 3
		“It was also the psychological side of things and I think that’s completely under-taken. It’s not taken into consideration at all. Because I tried to get it through the NHS and they wouldn’t possible, really. Luckily, I can pay for a private one, but it’s not… you get very desperate. It does affect your life if you go through any difficult times. The fact that you have something discharging constantly, it’s quite awful.” UK 8
Treatment	Surgical decision-making	“I suppose I’m just getting my head round the fact that the risks are not that much worse than living with a seton. They are worse but they’re not significantly worse. I’m now thinking it’s worth the gamble. Because with the seton obviously I’m not going to accidentally break wind in a public place when I’m giving a meeting or something. And so I didn’t want to risk that. But now I’m just going to have to… I’ve got my head round the fact that if that happens I’m just going to have to say I’m sorry I’ve got a bit of a condition and I’ll just have to just take it on the chin.” UK 1
	Frustration with healthcare system	“When we were going back in for dressing changes we kept saying he’s in a lot of pain, he’s got a temperature. And the registrar that was seeing us kept batting us away. And obviously at that point he was becoming really, really unwell. And so I think that maybe made the trust go a little bit.” UK 1
		“I know that obviously with Crohn’s and different sort of conditions they have specialist nurses and things and numbers that you can call for help and that but there’s absolutely nothing for this and I think the GPs in the nurses and GPs practices they don’t have a clue either because I’ve tried talking to them and yeah, it just that there’s so little known about it. Really little known.” UK 10
		“I understand that this fistula stuff most of the colorectal surgeons they want something more challenging. They want sort of cancer. They don’t really put a lot of energy and effort in this because it’s not like my local colorectal surgeons they’re very good at doing the type of stuff that they want to do cancer.” UK 2
	Aftercare	“But, that's where the second part follows. If you have surgery, there is virtually no aftercare… Look after such a procedure I personally think you have to go back every 14 days. To see how the wound is doing. This or that. But that's not going to happen. And that's a very big disadvantage.” NL 5
		“It’s all about the operation, it’s also about the aftercare, and that was completely left out with no… just dress the wound. Great, with what? And how, and expecting that the nurses will know how to do that. You know, we figure it out. We survived, but it was not ideal and I still wonder if the lack of knowledge would have affected my recovery. And after all, it was a failure.” UK 8
	Goals of treatment/healing	“My normal now or what I’d like to achieve is that I would like to just not really have to think about it. I’d just like to be able to go and exercise without thinking about, oh, am I going to be in pain? Can I actually do that? I don’t want to feel limited. I want to be able to just do stuff without really having to think about it.” UK 4
		“Get rid of it, I want it gone, I want to be back to normal. I want a little scar left on my bum cheek, which I’ve got to look in a mirror to see, and hopefully never look at or feel again. To me you got a branch, a tract running through your bum cheek and I just want it…I’d like it healed, I’d like it gone. So, I’m not in any pain, I’ve not got a second hole that comes out of me that shouldn’t be there and certainly not living with threads hanging out of me and it healed, you know that’s what I’d like.” UK 9

### Physical symptoms

#### Pain and discharge

The pain originated from the fistula itself and also as a result of treatment and dressing changes. Pain and discomfort varied in intensity, often described as ‘extreme’ or ‘debilitating’ at their worst. Although a separate symptom, discharge often contributed towards discomfort. Some described a cyclical nature to their symptoms, whereas others commented on the difficulty of managing their unpredictability.

#### Systemic symptoms

These would sometimes reflect an underlying infective process, manifesting as fever, chills and a ‘flu-like illness’ largely described prior to fistula diagnosis, or in the presence of a recurrent abscess. However, intense fatigue and tiredness were universal to all stages of the disease.

#### Abscess

Patients described having symptoms of an abscess prior to fistula diagnosis, with some describing recurrent episodes with an established fistula. Patients could recognise the presence of an abscess by increasing intensity of pain and a palpable swelling.

### Understanding the condition

#### Diagnosis

The period prior to diagnosis was often fraught with a lack of recognition of the underlying cause of symptoms, particularly when presenting to primary care. As a result, patients might have had several appointments or surgeries to drain abscesses prior to a definitive diagnosis. Patients described this period as confusing, experiencing relief when the underlying diagnosis was determined and other chronic illnesses were ruled out.

#### Information from healthcare professionals

The information given to patients was variable both between patients and within their individual experiences. Information regarding the nature of the fistula and treatment strategy was limited in the early stages of the disease, with some patients sensing reluctance from doctors to offer details. Experiences of good communication occurred in the tertiary setting, where patients spoke favourably of not feeling rushed, being given time to ask questions, and being given information specific to their own disease.

Patients felt that information about surgery, aftercare and signs of complications was lacking, and some found that hospitals and primary care gave conflicting advice. Patients particularly felt uninformed immediately after their surgery, where there was a lack of information regarding operative findings until subsequent follow up in the outpatient clinic.

Patients expressed that communication could be improved, particularly in complex cases. It was suggested that written communication and diagrams were helpful, but noted that the three-dimensional nature of the disease was challenging to comprehend.

#### Information gathering by patients

Patients frequently sought information regarding their diagnosis from online sources, particularly when information from healthcare professionals was conflicting or absent. Some specifically sought out surgical literature to increase understanding of treatment options to inform their decisions, especially when such detail was not divulged by their surgeon.

Practical and emotional support were found in online forums and social media groups. Learning from other patients' experiences and working through the emotional impact and practicalities of daily management meant that these communities were a highly valued resource and helped to fill a clear void.

#### Chronicity and complexity of condition

Understanding the full extent of disease was an ongoing process. Patients described a point of realisation where they recognised and accepted that there was no ‘quick fix’, and became aware that their recovery could be prolonged. There was frustration and disbelief that the disease could be so complicated and resistant to treatment.

### Life impact

#### Impact on work

Patients required significant time away from work as a result of undergoing investigations, treatment and recovery. This was measured in weeks rather than days and was amplified in patients with a delayed diagnosis or those who required multiple procedures. Aftercare had an additional disruptive impact when time was needed for regular dressing changes.

Patients noted that the direct impact of symptoms required them to make adjustments at work, such as avoiding prolonged periods of sitting, working from home and finding somewhere to change dressings. However, an indirect impact was also noted, due to being mentally pre-occupied with managing symptoms and fatigue affecting the ability to work efficiently.

Some patients found that the personal nature of the disease made it difficult to disclose to colleagues, using reasons such as back pain to explain physical adjustments and would endeavour to continue at work despite feeling unwell. For some, the fistula influenced long-term decisions, such as taking on freelance work, changing jobs and retiring early.

### Impact on daily living and activities

One of the significant impacts on daily life was the need for regular dressing changes. Patients reported needing a constant supply of equipment to do this and developed a routine around dressing changes, particularly when managing bowel motions and being away from home.

Daily adjustments were also required to manage fistula symptoms, such as finding a comfortable sitting or sleeping position and being mindful of what colour clothing to wear. Patients felt restricted in the range of activities they could participate in, and actively avoided doing things that would exacerbate their symptoms, such as bike- and horse-riding and swimming. Simple activities such as walking long distances and long car journeys would also be affected, having a more pervasive effect on social activities.

### Impact on relationships and intimacy

Often family and friends offered practical and emotional support, but a lack of understanding was noted regarding the chronicity of the condition, and limitations on travel and attending social gatherings meant that friends became distant over time.

Due to the intimate nature of the disease, patients spoke about the impact it had on their relationship with their partners. Patients expressed an increased dependency on their partner to provide assistance in managing the fistula, particularly when adjusting to new symptoms, hygiene demands and dressing changes in a body area that they could not visualise themselves. Patients felt guilty for its impact on their partner, who needed to make their own lifestyle adjustments around surgery and the recovery period.

Patients also spoke of difficulties with intimacy, largely due to a lack of desire and feeling that the fistula itself was a barrier to being intimate. Patients were highly conscious of the presence of fistula discharge, the smell and the need for dressings, which perpetuated feelings of shame and self-consciousness, resulting in little desire for sexual activity. For those who weren’t in relationships, the fistula and the perceived difficulty of explaining it to a potential partner were described as significant barriers to initiating relationships.

### Impact of treatment

Although surgical treatment provided hope of relieving symptoms and improving QoL, patients stated that treatment itself had emotional, mental and physical effects. In the immediate recovery, frustration at the prolonged period of inactivity was described, and patients were thankful for minimally invasive methods that resulted in rapid recovery time. Procedures requiring a large wound meant increased frequency and intensity of physical symptoms and further limitation of mobility and activity. Dressing changes were seen as highly inconvenient and the associated pain and discomfort were experienced as psychologically traumatic, particularly when dressing changes were required daily.

Multiple treatments had a cumulative effect on work and also in planning for life events such as holidays. Extra time away from work and special travel arrangements were required for follow up appointments with surgical teams, and travel to and from distant tertiary centres imposed additional time and financial burdens.

There was also mention of the actual impact of surgery on faecal continence. Anxieties were shared regarding the potential impact on long-term continence and whether repeated surgeries were going to result in the need for a stoma bag.

### Impact of seton

The impact of a seton was separate to that of other treatments, as its presence required additional adjustments. Some patients reported positive experiences, noting that their symptoms changed from being dominated by pain to ongoing discharge, which some found easier to manage, although this was not universal. A seton may have psychological benefits, particularly when it prevents abscesses. This was especially true for patients who had negative experiences of being unwell with an abscess. Those who had experienced a seton for a prolonged period of time had made necessary adjustments around it, and as a result, had reached a stage of seton acceptance.

In contrast the seton brought its own set of unique symptoms in addition to those from the fistula itself. It was frequently felt to be uncomfortable, and in the most extreme cases resulted in rubbing, chafing and skin blistering and further restriction of mobility. Managing the seton required additional adjustments, particularly when maintaining hygiene after bowel motions. Furthermore, patients had to be conscious of not pulling on the seton and several had experiences of the seton falling out, needing further surgery for replacement. Several patients spoke about the negative psychological impact of a seton, which was seen as unnatural and a clear deviation from normality. Persistent anxiety about keeping it clean and its impact on their intimate relationships was an additional psychological burden. These patients expressed fears of living with a permanent seton, and had difficulty accepting it as long-term treatment.

### Psychological impact

The psychological effects of having a fistula were wide reaching, and underlies many of the previous themes. The stress of managing physical symptoms drove a constant state of low-level anxiety. Patients would worry in anticipation of symptoms, particularly those who were fearful about developing an abscess. These patients had heightened anxiety around sudden and unexplained worsening of symptoms, leading to paranoia and frequent checking for any clinical signs of an abscess. The need to make daily adjustments to manage their symptoms required additional mental effort to plan for the day ahead in terms of activities they could tolerate, whether any additional dressings or equipment were needed, and what clothes it was ‘safe’ to wear. Patients described the mental exhaustion this caused, in addition to being a constant reminder that they were unwell.

Patients who had undergone multiple surgeries described the emotional toll this took. Recovery was painful and prolonged, and often represented a period of time in which there was uncertainty regarding the procedure and the eventual outcome. Inconsistencies in aftercare provided an additional stressor. Negative experiences were amplified for patients who later learned that surgery had been ineffective, where the prospect of further surgery and a repeat of these experiences resulted in more anxiety.

Whilst the psychological impact varied at different stages of the disease, feelings of uncertainty about the future, anxiety, frustration, embarrassment and isolation and low mood and depression were consistent in many accounts. Most reported the need for mental resilience to be able to process the psychological impact of an anal fistula, and some patients found professional help in the form of therapy and counselling highly valuable.

### Treatment

#### Surgical decision-making

Patients who had undergone several procedures became accustomed to the fact that no treatment was perfect, and reflected upon the thought processes that guided them to a specific treatment. Many described assessing the risks and benefits of a procedure, where the chance of relieving the worst aspects of their current symptoms were weighed against the worst outcome of treatment, of which incontinence and the need for a stoma were most frequently mentioned. Patients also noted that the weight they gave each of these scenarios changed over time, with some stating that they were more likely to choose low-risk options early on in their diagnosis. After repeated procedures, patients were more likely to consider higher-risk options if it meant a greater chance that their fistula could be cured.

#### Frustration with the healthcare system

Many patients commented on how the path to diagnosis was prolonged, with many barriers to accessing specialised care. In some instances, referral to secondary care was difficult to obtain. The wait for a specialist consultation was frustrating, particularly when this was followed by the need for more investigations. In some instances, patients cited poor communication and co-ordination between care providers, particularly for secondary and tertiary referrals. This was also demonstrated in patients who suffered from post-operative complications. These were monitored in primary care and only addressed by operating surgeons when the situation became an emergency or at formal follow up occurring much later. In some cases, dismissive attitudes expressed by doctors and surgeons, particularly when addressing concerns over complications, led to a mistrust of healthcare professionals.

Patients also felt that clinical expertise in and support for anal fistula was lacking. Several related the impression that the majority of surgeons were not practiced in the area and couldn’t offer specialised procedures. Patients expressed feelings of abandonment and frustration.

#### Aftercare

Patients spoke about a lack of comprehensive aftercare when recovering from surgery. They felt poorly prepared for managing their wounds or setons, were reassured that community services would direct their care, but found that even these providers had limited information and experience, and their practice could sometimes conflict with instructions given by the surgeon. The uncertainty of whether the wound was being cared for appropriately added to the psychological distress experienced in the recovery period, and some later reflected on whether inadequate aftercare contributed towards treatment failure.

#### Goals of treatment

Ideal outcomes from future therapies were mostly related to an absence of physical symptoms and physical reminders of the fistula, such as an external opening. This also included preservation of continence and the avoidance of permanent reminders of disease, such as a seton or a stoma. The importance of restoring QoL and ‘returning to normal’ was also emphasised. This meant restoring mobility and normal activities without considering the impact of the fistula or making adjustments for symptoms, dressings or painkillers. Patients who had experienced multiple surgeries explained how they were more likely to accept that the fistula wouldn’t be fully healed if it meant they could lead some semblance of a normal life.

## Discussion

The impact of having a cryptoglandular anal fistula expands far beyond physical symptoms. In research studies, impact on QoL is sometimes acknowledged as an outcome of intervention. Several studies have demonstrated that worsening faecal continence after anal fistula surgery results in deterioration of QoL scores [4, 7], however others have demonstrated that patients treated for simple fistula did not show any worsening of their Faecal Incontinence Quality of Life (FIQL) scores despite showing minor worsening of continence [10]. Data obtained from our interviews demonstrates the complexity of making this risk-benefit assessment, where some patients who experience the worse end of the spectrum of fistula symptoms would take bigger risks in treatment, including impairment of continence, if it meant relief from their fistula. Owen et al. found that fistula patients demonstrated impaired QoL on all domains of the Short Form-36 (SF-36) questionnaire compared to the general population [4] and along with other studies, have shown that patients with recurrent disease had worse QoL compared to those with a primary fistula [4, 15]. However, overall QoL as measured with the Gastrointestinal Quality of Life Index (GIQLI) did not differ between patients with a fistula and age-matched controls [16]. Furthermore, there is suggestion that people with a prolonged disease course exhibit better QoL scores than those with a shorter time with clinical symptoms due to the extensive adaptations made around the disease [17]. Adjustment around symptoms was seen in many of our sub-themes, including in activities of daily living, adjustments made at work and around a seton, with participants essentially describing a ‘new normal’ way of living. In summary, although previous studies have shown variation of QoL and alluded to the broad themes identified in this study [8, 18, 19], to our knowledge this is the first in-depth exploration of what it means to live with a cryptoglandular anal fistula. The qualitative techniques we have employed have allowed us to identify how these themes are inter-related, for example, how physical symptoms require readjustment of daily activities and working patterns, the negative impact this has on psychological wellbeing and the additive effect of these additional stressors on managing an unpredictable illness, providing some explanation as to why other studies sometimes see conflicting outcomes.

A similar qualitative investigation has been conducted in patients living with a Crohn’s anal fistula, finding similar themes [11]. However, there are subtle differences in that the side effects of long-term medication addressing the underlying inflammatory nature of Crohn’s fistulae were a significant concern for patients with Crohn’s Disease, who also cited a loss of independence due to requiring regular infusions in hospital. Apart from this, the negative psycho-social effects were similar in both diseases.

The investigation into patient experience prior to diagnosis and throughout the treatment journey has highlighted important issues regarding clinical management. A common theme throughout all interviews was dissatisfaction with the information, support and aftercare given by healthcare providers. The presentation of anorectal abscesses can be insidious and is not always associated with a fistula. However, patients reported that doctors failed to investigate incomplete wound healing or recurrent abscesses, requiring them to take ownership of their own care to ask for specialist referrals and further investigations. Even within secondary care, patients expressed frustration that the information and treatment options provided seemed limited, and felt that cryptoglandular disease was given less emphasis in healthcare provision. Furthermore, inconsistencies in aftercare and a lack of feedback from surgeons fueled the uncertainty that patients experienced, which contributed to the negative psychological impact of treatment and recovery.

Previous studies have shown how patients and clinicians have different priorities when it comes to the outcome of fistula treatment [8]. Whilst patients reported wanting an improvement in the physiological aspects of the disease, there was also a significant focus on reducing the adjustments required to manage their fistula and the psychological impact of constantly thinking about and planning a routine around their symptoms. This suggests that there is some dissonance between clinician and patient expectations of treatment, as judged by the most frequently measured outcomes in interventional studies [3].

Taken together, these findings suggest that changes are required in clinical practice and future research to improve care for these patients. First, improvement in clinical practice needs to be driven by greater education amongst healthcare professionals within all care settings regarding the diagnosis and management of cryptoglandular anal fistula, to ensure that patients receive a timely diagnosis and are referred for further investigations and treatment appropriately. This should be expanded to include allied healthcare professionals and nursing staff involved in providing wound and post-procedural care for patients, to ensure that best clinical practice is followed and that patients are reassured with continuity of care. Furthermore, an emphasis on providing emotional and psychological support should be made, ensuring a more holistic approach to fistula treatment. Second, if the complexity of the disease is beyond the scope of practice within one centre, referral to a specialist centre, with a wider range of treatment strategies and access to experimental therapies may be appropriate. In addition, patient experience of fistula treatment can be greatly improved by ensuring they receive adequate, easy to understand and complete information regarding the extent of disease and operative management in a timely manner following surgery as well as consistency in wound care advice. With regards to future research directions, greater emphasis on the nature and chronicity of cryptoglandular perianal disease is needed, as well as studies that work towards clarifying optimal management pathways, addressing the heterogeneity of current clinical practice [20, 21]. Finally, the patient experience of disease and desired outcomes, particularly the impact on QoL should receive greater emphasis in the design and methodology of future research studies, ensuring that the outcomes that are most valued by patients are given due consideration.

There are some limitations to this study. All patients were recruited from tertiary referral centres, meaning that disease will naturally be more complex, and one of the issues raised by participants was the prolonged time to diagnosis and treatment, which may be specific to this particular group. As a result, some early or less impactful disease related issues could have been overlooked in our study and may require further, focused investigation. However, we were able to include some participants who had not yet undergone treatment and some symptoms, and therefore the impact on patients, would be similar to those with simple fistulae attending primary or secondary care, regardless of duration of disease. Furthermore, our findings have value in identifying the issues that occur prior to diagnosis and in the immediate post-operative period, which would be common to all patients. The youngest patient in our sample was 30 years old, and so our findings may not be generalisable to the youngest age groups who suffer from this condition, and the impact on areas such as educational attainment could have been overlooked. Finally, we conducted this study across two different countries which may vary in cultural attitudes and access to healthcare services, which could influence patient perceptions of illness and disease. Whilst the aim of this study was to provide a general overview of patient experience rather than to investigate cultural differences, we accept that this would be an interesting area of future research. However, this should be investigated using a dedicated study with questioning focused on this aim and an adequate sample of patients from different cultural groups and countries. Despite this, we have conducted a detailed and extensive exploration of cryptoglandular anal fistula, which has not been reported before.

## Conclusion

Cryptoglandular anal fistula has an extensive impact on all aspects of daily living and a consequent effect on psychological and emotional wellbeing. Patients frequently felt that information and treatment options for their condition were limited. The ultimate aim of treatment for many was to be able to live as normal a life as possible. This suggests that future studies of fistula treatment should have a more holistic focus on outcomes and individualised patient goals. Clinicians can address deficiencies in care by providing clinical and practical information, timely referral where there is limited experience of disease and direct patients towards helpful resources such as online support groups and sources of psychological support.

## Supplementary Information

Below is the link to the electronic supplementary material.

Supplementary file1 (DOCX 25 kb)
