# Supplementary material for: Living with cryptoglandular anal fistula: a qualitative investigation of the patient's experience through semi-structured patient interviews
Source: Qual Life Res. 2022 Feb 17;31(8):2505–18. doi: 10.1007/s11136-022-03098-y (PMC9250474; doi:10.1007/s11136-022-03098-y)
Supplement: Supplementary file 1 — Supplementary file1 (DOCX 25 kb) [file 11136_2022_3098_MOESM1_ESM.docx]

Appendix 1: Interview guide

1 – Introduction

- Go over the purpose of the study
- Check willingness to take part in the study
- Check if participant agrees that the interview be audio-recorded, stored and used for presenting research findings anonymously.
- Check if there are any questions regarding the interview procedure
- Ask participant to complete the informed consent form if not already completed

2 – Patient experiences of living with a fistula

1. **Ask about the experience of having cryptoglandular anal fistula**
   1. ‘I understand you have (had) a fistula. Can you tell me more about that?’
   2. ‘When did your problems with the anal fistula begin?’/‘Could you tell me about how you first found out you had a fistula?’
   3. ‘What type of inconvenience did you have after being diagnosed with a fistula’
   4. ‘Can you tell me what life with a fistula is like for you?’/’What is the impact on daily life?’/’Did it impact on your social life?’/’Did it impact on your sex life?’
   5. ‘What do you feel is the worst aspect of having a fistula?’
   6. ‘How do you manage the fistula?’
2. **Ask about the treatment that was offered and how they decided about undergoing treatment**
   1. ‘What treatment(s) were you offered and which did you choose/ receive?’
   2. ‘Were you aware of the pros and cons of the different treatment options?’
   3. ‘What made you decide onone treatment over the other?’/’What factors did you consider in deciding on a treatment?’
3. **Ask about the effects that treatment had/is having**
   1. ‘Was your treatment successful, and why?’/’How did the treatment impact on daily life/ working life/ social life/sex life?’/’How did you notice that the fistula treatment worked (if it worked)?’
   2. ‘Did you experience any negative effects right after treatment or later on?’
   3. ‘How bad were these negative effects? Would you choose this treatment again or recommend it to a family member?’
   4. ‘What do you feel is the worst aspect of the treatment?’
   5. ‘What would you say is the most important result after the treatment?’
   6. ‘Did your perspective on what is important change over time?’
   7. ‘Overall, were you satisfied with the results of the treatment? Did the treatment match your expectations?’
4. **Ask about concerns for the future**
   1. ‘Do you have any concerns for the future? What is that you are most concerned about? Has this changed over time?’

3 – Outcomes believed to be relevant and important

1. **Ask about important results of treatment**
   1. In future, we want to be able to select a treatment that best suits the patient’s preferences. Based on your experience, ‘what would be the most important outcome/result of the treatment?’ ‘What should treatment achieve/ what are your ultimate goals of treatment?’

4 - Closing

1. **Ask whether there is anything else they would like to talk about**
2. **Check if there are any questions**
